# Supplementary material for: Umbilical Cord Tensile Strength Under Varying Strain Rates
Source: Bioengineering (Basel). 2025 Jul 22;12(8):789. doi: 10.3390/bioengineering12080789 (PMC12382923; doi:10.3390/bioengineering12080789)

Supplementary Figure S1. Stress-strain curve for specimen D01, annotated to highlight key mechanical parameters: maximum load, elongation at maximum load, elongation at break, and stiffness. These parameters provide insight into the material's strength and deformation characteristics. Stress is reported in megapascals (MPa), and strain is given as the dimensionless ratio of elongation to the initial length.

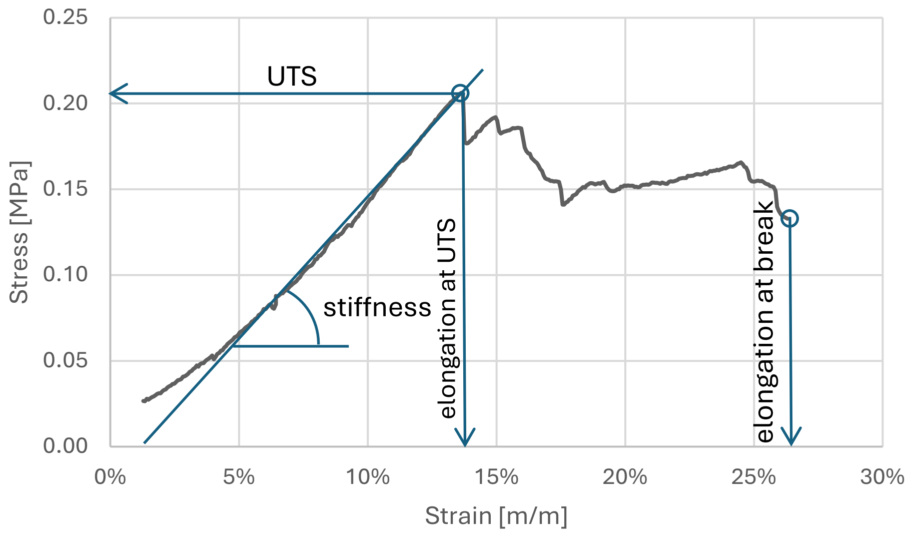

Supplementary Figure S2. Representative stress-strain curves corresponding to the three analyzed strain rates are shown. The initial portion of each curve exhibits a linear relationship between stress and strain, indicative of elastic behavior. Upon reaching the peak load, the material response becomes nonlinear and more erratic, with the post-peak slope (softening behavior) varying as a function of the applied strain rate. Stress is reported in megapascals (MPa or N/mm^2), while strain is expressed as the ratio of elongation to initial length (mm/mm, dimensionless).

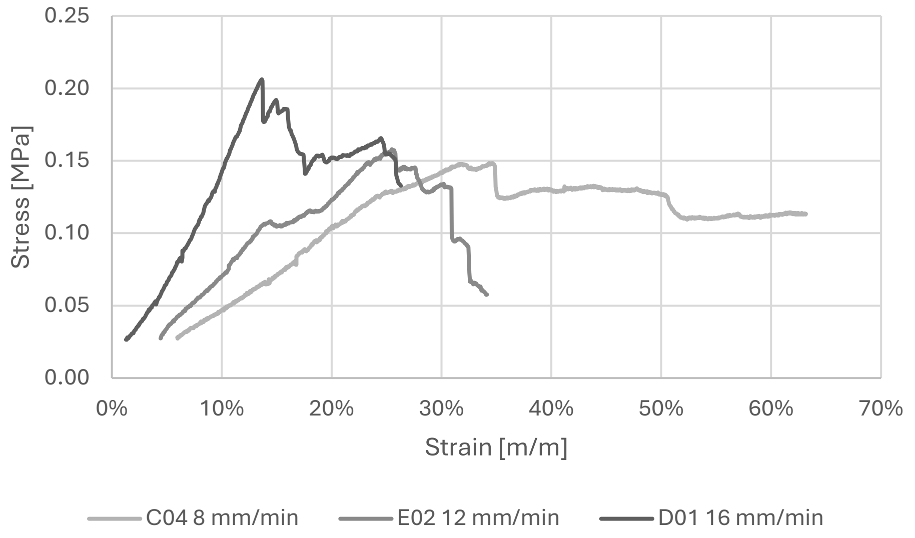

Supplementary Figure S3. The Standard Linear Solid (SLS) viscoelastic model response to a constant stress (left) or strain (right) in a time-dependent manner for composite material. This creep behavior is characterized by the transfer of stress from the matrix to the fibers, resulting in an increase in fiber strain that matches the overall strain of the composite.

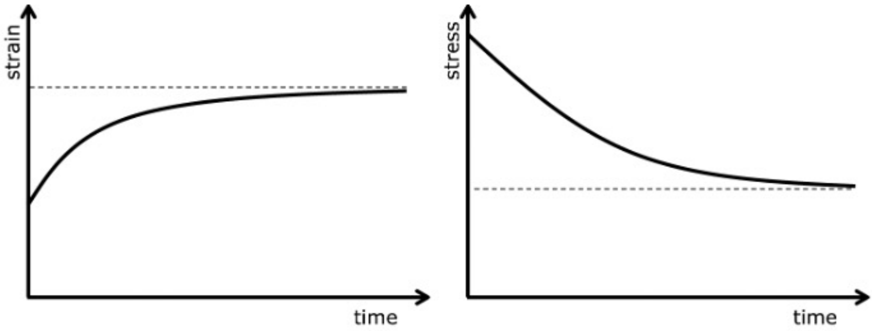

Supplement: Supplementary file 1 [file bioengineering-12-00789-s001.zip › Supplementary Figures.pdf]
